# Diagnosis and management of neurofibromatosis type 1 in Arabian Gulf Cooperation Council Region: challenges and recommendations

**DOI:** 10.3389/fonc.2024.1323176

**Published:** 2024-08-27

**Authors:** Fahad A. Bashiri, Khaled Hundallah, Musaad Abukhaled, Mossaed Mohammed Alyahya, Amna Al Futaisi, Daniah Alshowaeir, Asmaa Al Tawari, Shaker Abdullah, Ata Ur Rehman Maaz, Eman Taryam AlShamsi, Walaa Alshuaibi, Faisal Alotaibi, Hesham Aldhalaan

**Affiliations:** ^1^ Division of Pediatric Neurology, Department of Pediatrics, College of Medicine, King Saud University Medical City, King Saud University, Riyadh, Saudi Arabia; ^2^ Prince Sultan Military Medical City, Riyadh, Saudi Arabia; ^3^ Neuroscience Centre, King Faisal Specialist Hospital and Research Centre, College of Medicine, Al Faisal University, Riyadh, Saudi Arabia; ^4^ Consultant of Neuro-oncology and Neuromuscular-neurology, Department of Oncology, King Faisal Specialist Hospital and Research Centre, Department of Neuroscience, King Faisal Specialist Hospital and Research Centre, Al Faisal University, Riyadh, Saudi Arabia; ^5^ Sultan Qaboos University, College of Medicine and Health Sciences, Muscat, Oman; ^6^ Department of Ophthalmology, College of Medicine, King Saud University, Riyadh, Saudi Arabia; ^7^ Pediatric Neurology Unit, Pediatric Department, AlSabah Hospital, Ministry of Health, Kuwait City, Kuwait; ^8^ Department of Oncology, King Faisal Specialist Hospital & Research Center – Jeddah (KFSHRC-Jed), Jeddah, Saudi Arabia; ^9^ HemOnc Division, Department of Child Health, Sidra Medicine, Doha, Qatar; ^10^ Pediatric Hematology-Oncology Department, Al Jalila children’s specialty Hospital, Dubai, United Arab Emirates; ^11^ Division of Medical Genetics, Department of Pediatrics, King Khalid University Hospital, College of Medicine, King Saud University, Riyadh, Saudi Arabia; ^12^ Neuroscience Centre, King Faisal Specialist Hospital and Research Centre, Riyadh, Saudi Arabia; ^13^ Department of Neurosciences, King Faisal Specialist Hospital & Research Centre, Riyadh, Saudi Arabia

**Keywords:** children, Gulf Cooperation Council, management, neurofibromatosis type 1, referral

## Abstract

Neurofibromatosis type 1 (NF1) is a complex multisystem genetic disorder that requires long-term, age-specific monitoring and multidisciplinary care. NF1 symptom burden can significantly affect the quality of life and impose a substantial economic burden on patients and their families. The approval and widespread availability of mitogen-activated protein kinase (MEK) inhibitors such as selumetinib for NF1-related plexiform neurofibromas have revolutionized the standard of care for patients with NF1, however their effective utilization hinges on early recognition of NF1. We present a consensus manuscript describing the challenges observed in the Arabian Gulf Cooperation Council (GCC) for diagnosing and managing NF1. Experts from the GCC also present recommendations for the early recognition and management of NF1 and its complications. A referral pathway that can play a crucial role in helping primary healthcare providers refer their patients to experts is also proposed. Increasing the availability and accessibility of genetic testing at an affordable cost and optimizing personalized NF1 care are essential for NF1 management. Developing regional guidelines for NF1 management and establishing NF1 centers of excellence may facilitate better care and outcomes for patients with NF1 in the GCC region.

## 1 Introduction

Neurofibromatosis type 1 (NF1) is a progressive genetic disease, with a generally estimated global prevalence of 1 in 3000 to 1 in 6000 people (1, 2). Recent studies have shown that NF1 is more common with a birth incidence of 1 in 2000 (3) and prevalence of 1/4088 (95% confidence interval (CI) 1 in 3,869 to 1 in 4,320) (4). It is characterized by neuroectodermal anomaly that mainly affects the skin, nervous system, eyes, bones, and possibly other organs, causing significant morbidity or mortality (1, 2). NF1 is caused by heterozygous mutations in the neurofibromin gene (17q11.2) (5), which exhibits a high mutation rate of one in about 10,000 alleles per generation (6). This mutation is nearly 100‐fold higher than the average mutation rate per locus per generation (6).

Nearly 33% to 50% of NF1 cases are caused by sporadic mutations (2, 6). Several studies have found NF1 genotype-phenotype correlations and provided an overview of their significance, advocating their role in improving the management and genetic counselling of patients with NF1 (7–9). Koczkowska reported a genotype-phenotype correlation at the NF1 region 844–848, indicating a higher predisposition to malignancies in these individuals compared to the general NF1-affected population (p = 0.0061) (10). Patients with microdeletions in the NF1 gene were reported to have a 4-fold increased risk of malignant peripheral nerve sheath tumors (MPNST) and this risk was further increased with codeletion of SUZ12 or EED gene (11). Therefore, diagnosis based on the clinical features and genetic testing may help to provide better care for patients with NF1 (7). Data is very limited on genotype-phenotype correlations in NF1. Although more than 3000 genetic mutations have been reported in the NF1 gene, only 4 actionable genotype-phenotype correlations are reported — type 1 microdeletion (caused by interchromosomal nonallelic homologous recombination), type 2 microdeletion (involving 1.2 Mb, linked with hemizygosity of 13 protein-coding genes), type 3 microdeletion (involving 1.0 Mb, linked with hemizygosity of 9 protein-coding genes), and type 4 microdeletions (unusual, comprised of a variable number of genes in the deleted region) (11). In most cases, disease severity is not typically predicted by a specific mutation and disease manifestations may vary within families carrying the same mutation (12). Therefore, a patient-centered longitudinal care with age-specific monitoring of clinical manifestations can be helpful for the early recognition and symptomatic treatment of disease sequelae in patients with NF1 (1). Due to the complexity of the disease, a multidisciplinary approach with frequent engagement between specialized healthcare providers (HCPs) in timely referral may improve the quality of care in patients with NF1 (13). The purpose of this consensus manuscript is to provide recommendations on the diagnosis, referral, and management of patients with NF1 for HCPs. This manuscript will also provide guidance for setting up the NF1 clinic and NF1 center of excellence in the Arabian Gulf Cooperation Council (GCC) region.

## 2 Methodology

We planned a 3-step process to formulate the consensus recommendations for diagnosing and managing NF1 in the GCC region. A pre-meeting questionnaire-based survey was conducted focusing on disease burden, diagnostic, and management practices for NF1 in the GCC region (**Supplementary Material**). A literature search was conducted using PubMed to gather relevant publications on NF1 in the GCC region and internationally. A pre-meeting questionnaire based on the gathered information was formed and later validated by three of the Saudi Pediatric Neurology expert members. This questionnaire was disseminated to a group of 13 experts (from Saudi Arabia, Kuwait, UAE, Oman, and Qatar) formed by the Saudi Pediatric Neurology Society as an internal survey. The expert committee meeting convened on December 10, 2022, included a panel comprising a pediatric neurologist and oncologist, neuro-oncologist, neuro-ophthalmologist, neurosurgeon, and medical geneticist, which was supported by AstraZeneca GCC. The panel discussed the available data on disease burden and provided an update on the diagnostic and management practices and associated challenges specific to their region. Additionally, panel members recommended developing a referral algorithm and setting up NF1 clinics and/or NF1 centers of excellence in the GCC region. We present a consensus manuscript with recommendations for diagnosing and managing NF1 in the GCC region based on an evidence-based literature search and expert opinions. All experts critically reviewed, revised and approved the manuscript.

## 3 Disease burden in GCC

A high proportion of the Arab population is affected by genetic disorders, accounting for 451 genetic disorders in the combined Arab populations of Bahrain, Oman, and the UAE; Oman with the most number of genetic disorders (14). Factors such as a higher prevalence of consanguineous marriages (Saudi Arabia [58%], Kuwait [54%], UAE [50%], and Qatar [52%]), lack of public health initiatives for preventing genetic and congenital abnormalities, inadequate prenatal and postpartum care and restriction on services to prevent and control of genetic disorders are accountable for higher prevalence of genetic disorders in GCC region (14–17). Patients with NF1 have been reported to be at an increased risk of developing malignancies and have a life expectancy of nearly 10 to 15 years shorter compared to the general population (18, 19). Accurate data on the incidence and prevalence of NF1 in the GCC region along with the burden of the disease are not available. In addition, there is a lack of regional NF1 registries in the GCC region; most studies have evaluated the clinical spectrum and comorbidity associated with NF1 or the correlation between a specific genotype and phenotype (20–22). Based on the data of the pre-meeting questionnaire, the average number of cases of NF1 seen every month by the majority of the experts was ≤10. Apart from the clinical burden, NF1 is linked with the physical, social, economic, and psychological burden with each having a decent share in affecting the patient’s quality of life. All experts believed that amongst all of them, the psychological burden has the highest impact on the patient. A qualitative content analysis study by Foji et al., also reported a high level of both psychological and social burden on patients with NF1 that caused mental conflicts, eventually resulting in delayed developmental milestones among these patients (23).

## 4 Clinical presentation and diagnosis of NF1

### 4.1 Clinical diagnosis

Since 1987, the diagnostic criteria established by the National Institutes of Health (NIH) have been employed for clinical diagnosis of NF1 (**Supplementary Table 1**) (24–27). In 2021, Leguis et al. presented an international consensus recommendation with revised diagnostic criteria, incorporating new clinical features and introduced the possibility of genetic diagnosis for NF1 diagnosis (**Table 1**) (26). Café-au-lait macules (CALMs) are the most frequent and innocuous disease manifestations of NF1, followed by axillary freckling, neurofibromas, and Lisch nodules along with NF1-specific disease complications (25). Experts from the GCC region reported that CALM followed by optic pathway tumors are the most commonly seen disease manifestations of NF1, consistent with a finding of several studies that CALM is the most frequent NF1 disease manifestation (22, 28, 29). An observational retrospective cohort study from Saudi Arabia that included 50 patients with NF1 demonstrated that 90% of patients had CALM; 34% had skinfold freckling and 24% had at least one or more plexiform neurofibromas. Furthermore, 42% of the patients had a first-degree relative with NF1, and approximately a quarter had epilepsy. Nearly 90% of the neuro-radiological characteristics were consistent with those of NF1, 52% of the patients had one or multiple types of tumors, and 34% had optic pathway glioma and were malignant (22). Nearly 19% of the patients with NF1 had endocrine diseases, of which the majority had short stature followed by subclinical hypothyroidism, underscoring the need for screening for endocrine abnormality in patients with NF1 to minimize complications and the late presentation should be considered (30).

**Table 1:** Revised diagnostic criteria for NF1.

A: The diagnostic criteria for NF1 are met in an individual who does not have a parent diagnosed with NF1 if two or more of the following are present	
CALM	Six or more, >5 mm in greatest diameter in prepubertal individuals and > 15 mm in greatest diameter in postpubertal individuals
Freckling	Axillary or inguinal regions^a^
Neurofibromas	Two or more neurofibromas of any type or one plexiform neurofibroma
Optic pathway glioma	Presence
Lisch nodules/choroidal abnormalities	Two or more iris Lisch nodules identified by slit-lamp examination or two or more choroidal abnormalities—defined as bright, patchy nodules imaged by optical coherence tomography (OCT)/near-infrared reflectance imaging
Distinctive osseous lesion	Sphenoid dysplasia,^b^ anterolateral bowing of the tibia, or pseudarthrosis of a long bone
*Genetic risk*	A heterozygous pathogenic NF1 variant with a variant allele fraction of 50% in apparently normal tissue such as white blood cells
B: A child of a parent who meets the diagnostic criteria specified in A merits a diagnosis of NF1 if one or more of the criteria in A are present	

CALM, café-au-lait macules; NF1, neurofibromatosis type 1. Adopted from Leguis et al., 2021 (26).

a If only CALM and freckling are present, it is most likely to be a NF1 diagnosis, but in rare cases, the person may have a different diagnosis like Legius syndrome. There should be bilaterality in at least one of the two pigmentary findings (café-au-lait macules or freckling);

b Sphenoid wing dysplasia is not a separate criterion when there is an ipsilateral orbital plexiform neurofibroma.

**Figure 1:**
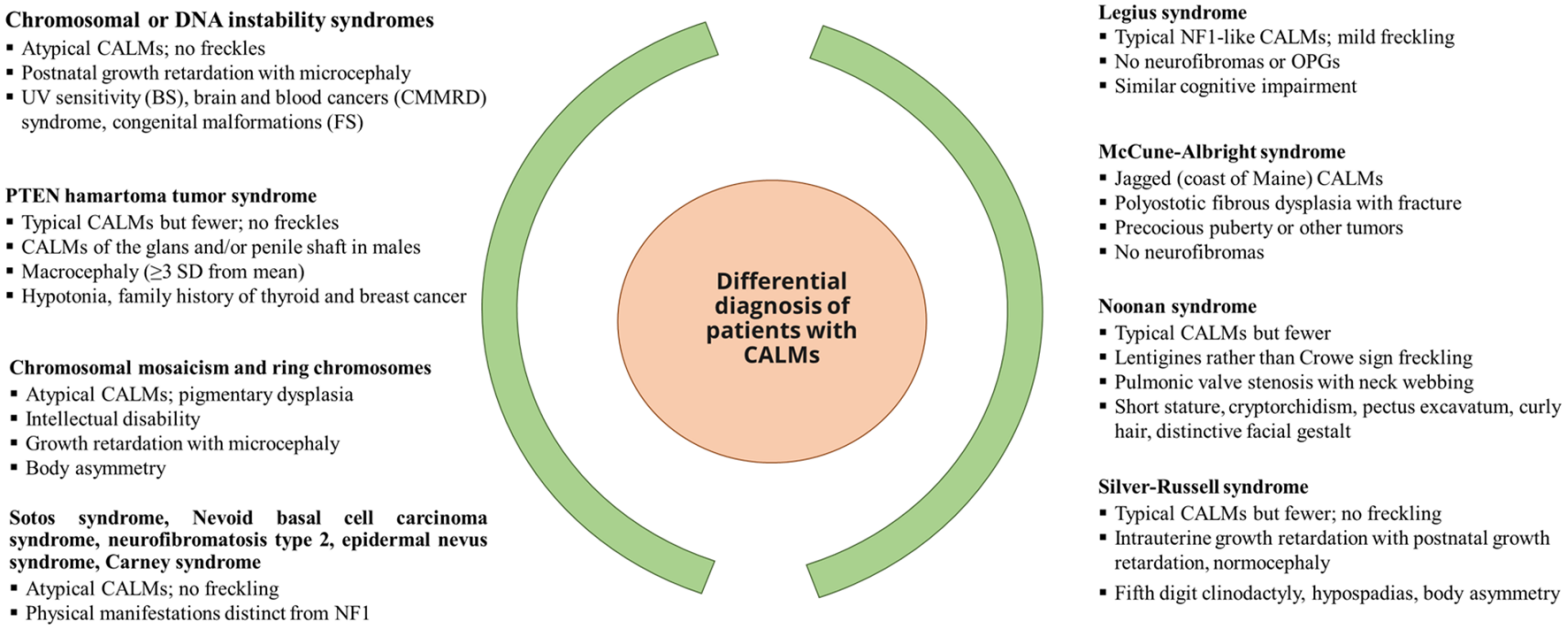
Differential diagnosis of patients with CALM. [Source: Miller et al, 2019 (35)]. BS, Bloom syndrome; CALM, café-au-lait macules; FS, Fanconi syndrome; CMMRD, constitutional mismatch repair deficiency; OPG, optical pathway glioma; PTEN, phosphatase and tensin homolog; UV, ultraviolet.

Nearly 46% of sporadic cases of NF1 fail to meet the NIH diagnostic criteria by the first year of age, resulting in a delay in NF1 diagnosis (25). The NF1 diagnosis in non-familial pediatric patients may be difficult due to the presence of certain age-dependent clinical manifestations along with the variability in NF1 clinical expression, which makes it difficult to predict future manifestations in affected children with NF1. Other conditions such as Noonan syndrome and cardiofaciocutaneous syndrome have overlapping phenotypes with NF1 (31). A distinctive natural histories have been recognized [such as segmental/mosaic NF1 (32) and constitutional mismatch repair deficiency syndrome (33)] alongside the identification of new probable criteria for NF1, such as nevus anemicus (34). **Figure 1** presents the overview of the differential diagnosis of patients with CALM.

### 4.2 Genetic testing

NF1 is an autosomal dominant genetic disorder, showing a 100% penetration, which does not skip generations or has asymptomatic carriers (1, 36). The likelihood of a NF1 parent having a child with NF1 is 50% (1, 35). An unaffected parents of a child with a new pathogenic sequence variant have a lower risk of recurrence in siblings of the child with NF1 (35). A detailed family history must be obtained in pediatric patients for NF1 diagnosis. Most of the experts from GCC agreed that they use genetic and clinical criteria for diagnosing NF1; half of the experts referred >50% of their patients for genetic testing. A few of the experts agreed to use clinical criteria for diagnoses due to the lack of genetic testing facilities available at some centers. Other barriers reported were higher cost and inaccessibility of the genetic testing facilities for NF1 (37), which may also limit the use of genetic testing in the GCC region. Bergqvist et al. suggested that most of the HCPs use NIH clinical criteria for NF1 diagnosis without performing genetic testing, especially in cases of established clinical diagnosis (1). However, genetic testing can help diagnose patients with an unusual phenotype, or atypical clinical presentation (32) or for differential diagnosis of NF1 from other overlapping conditions such as Legius syndrome and Noonan syndrome (31, 38).

Leguis et al. demonstrated a very high consensus regarding the inclusion of genetic diagnosis, without indicating the necessity or recommendation of genetic testing for NF1 diagnosis (26). Genetic testing also aids in referring parents for genetic counseling with respect to prenatal screening and any future pregnancy. In view of the published literature, data from the pre-meeting questionnaire, and experts’ opinions, the panel suggested incorporating clinical criteria and genetic testing-based diagnosis for NF1 based on its availability and affordability.

## 5 Principal NF1 manifestations

### 5.1 Dermatological manifestations

Dermatologic manifestations of NF1 CALM, skinfold freckling, and neurofibromas. A real-world study comprised of 1102 patients with NF1 (1983 to 2020) reported that 96.5% of the patients had CALM; 90% had axillary and inguinal freckling and 78.1% had neurofibromas (39). Nearly 83% to 99% of patients had CALM at birth (20, 25, 40). CALM and a positive family history of NF1 are the most common reasons for the initial assessments (41). Skinfold freckling is another dermatological manifestation of NF1, affecting >80% of NF1 individuals (29). Axillary and inguinal freckles can appear in any area where skinfolds are in apposition, typically beginning at about 3 to 5 years of age (35). Additional dermal manifestations include juvenile xanthogranulomas (small, waxy, yellowish nodules that appear in the skin of a small percentage of young children with NF1) (35, 42), nevus anemicus (flat skin macule, paler than surrounding skin and occur in up to 50% of the NF1 individuals) (35, 43) and pruritus (35).

Neurofibromas are benign peripheral nerve sheath tumors that include cutaneous (dermal), subcutaneous (peripheral nodular neurofibromatosis), and internal (nodular and plexiform neurofibromas). Although cutaneous neurofibromas are benign without any risk of malignancy, they may impact the quality of life in patients with NF1 (1, 40). Subcutaneous neurofibromas affect nearly 20% of the NF1 cases (44) and can result in neurological deficits or tingling along the affected nerve (1). The internal neurofibromas are related to a high-risk phenotype and may result in the MPNST development. Therefore, careful clinical monitoring and sequential whole-body MRI (WB-MRI) are needed for the early diagnosis and treatment of MPNSTs in high-risk patients (45, 46). Among dermatological manifestations of NF1, this paper primarily focuses on plexiform neurofibromas due to the associated increased morbidity and mortality in patients with NF (47).

### 5.2 Plexiform neurofibromas

Approximately 27% to 56% of patients are affected by plexiform neurofibromas (46, 48, 49), which can vary location along the nerve, may be superficial, or grow into a complex deep mass that involves multiple nerves (40, 47). Deep lesions can cause disfigurement and dysfunction by infiltrating structures like fascia, muscle, or more internal components of the body (47, 48). Regional or WB-MRI is the imaging modality of choice for identifying and characterizing plexiform neurofibromas. Studies have shown that 47% to 56% of the patients with NF1 had invasive plexiform neurofibromas (46, 48, 50). Mautner reported that 52% of invasive plexiform neurofibromas were found in the face, head, and neck area during WB-MRI (50). Most plexiform neurofibromas are diagnosed in children before 5 years of age (48).

### 5.3 Malignant manifestations

#### 5.3.1 Malignant peripheral nerve sheath tumors

MPNSTs are aggressive sarcomas that may develop in 8% to 16% of patients with NF1 (1, 51), frequently transforming from nodular or plexiform neurofibromas mainly after puberty (40). The rapid tumor growth with pain or the development of new neurological symptoms must alert the HCPs for MPNST (1, 52). Factors associated with poor MPNST prognosis are the presence of more than one primary tumor (53), large tumor size (53–55), located at the axial/trunk region (52, 54), high histological grade (54), telomerase activity and overexpression of telomerase reverse transcriptase (56) and genomic alterations in chromosomes 10, 16, and Xq (57). The multidisciplinary team (MDT) approach must be used for the evaluation and management of suspected cases of MPNST and for choosing an effective strategy for biopsy and treatment.

#### 5.3.2 Optic pathway gliomas

Nearly 15–20% of pediatric patients with NF1 have optic gliomas (58), which are typically indolent tumors that frequently develop within the optic pathway before the age of 8 years (59). The majority of optic pathway gliomas remain indolent without affecting the patient’s vision; but, in a few patients, they can result in vision loss and other morbidities such as precocious puberty (35, 60).

### 5.4 Neurological manifestations

Patients with NF1 are more prone to develop headaches frequently, particularly common migraine headaches, and seizures compared to the general population (61, 62). The increased risk of headaches and migraines may be attributed to increased intracranial pressure, visual disturbance, and structural brain lesions in patients with NF1 (35, 63). The most commonly occurring neurological manifestations are developmental delay, learning disabilities, visual signs, and attention-deficit hyperactivity disorder (predominantly inattentive) (41). Salman et al. reported that patients with attention-deficit hyperactivity disorder had lesser focal abnormal signal intensities (FASI) at baseline MRI and it was more in cases when a first-degree relative(s) had NF1 or decreased visual acuity, suggesting the number of FASI may be correlated with some clinical features (41). Seizures are usually focal, and occur at any age; however, a focal central nervous system lesion needs attention and prompt brain MRI is recommended at presentation with a new onset of a seizure (35). When a child exhibits delays in psychomotor and/or language development, the physician must promptly refer them to the appropriate professional for early intervention and management (1). Patients must be referred to a neurologist in case of frequent headaches that are not controlled by over-the-counter medications (35).

### 5.5 Orthopedic manifestations

NF1-related skeletal abnormalities in children with NF1 include macrocephaly, osteomalacia, osteopenia, and localized bone dysplasias (35, 64). Studies have reported that nearly one-third of children have postnatal growth delay and slightly reduced pubertal growth (35, 65), 37.5% have macrocephaly (66) and 7.2% have congenital dysplasia of the long bones (67). Scoliosis, is another common orthopedic manifestation in patients with NF1, affecting 10% to 28% of the patient population (1, 68). Dystrophic scoliosis is frequently associated with vertebral dysplasia; reported in 59.3% to 70% of patients with NF1 on whole spine MRI (1, 69). Patients with NF1 are also at increased risk of having impaired mineralization, such as osteopenia (48%) and osteoporosis (25%) (70, 71).

### 5.6 Endocrine manifestations

The most common endocrine disorders in NF1 are short stature with or without growth hormone deficiency, central precocious puberty, and growth hormone excess (72). In NF1, gynecomastia and diencephalic syndrome alongside the presence of endocrine tumors like pheochromocytoma are additional common endocrine-related conditions (72). In view of NF1-related puberty manifestations, adolescent children must be assessed for abnormal growth acceleration or early development of secondary sexual characteristics that may be linked to an optic pathway glioma involving the chiasma (73, 74).

## 6 Evidence-based and panel recommendations for the management of NF1

**Figure 2:**
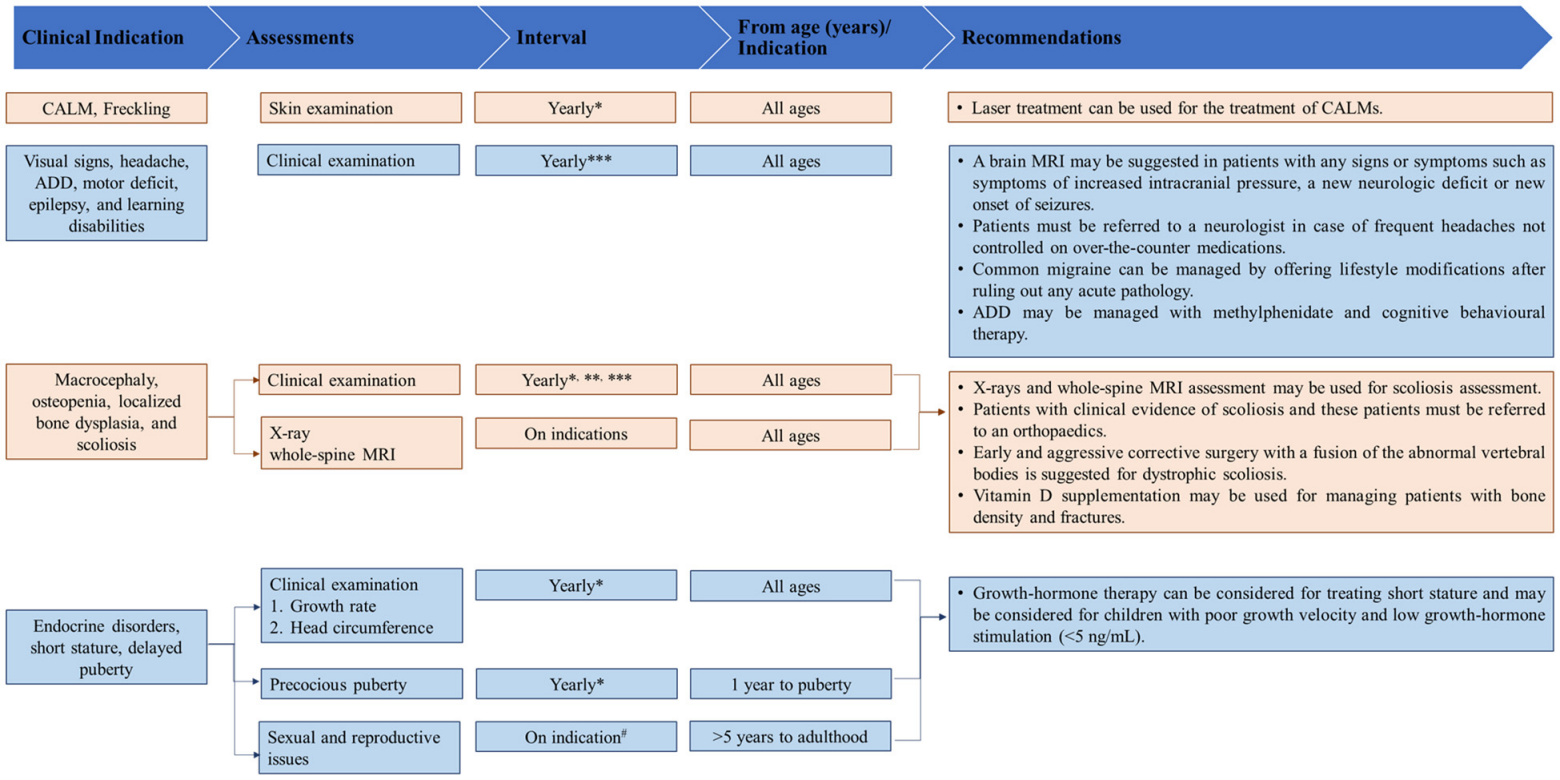
Overview of frequency of clinical and developmental assessments in patients with NF1. [Source: Bergqvist et al, 2020 (1); Miller et al., 2019 (35)]. ADD, attention-deficit disorder; MRI, magnetic resonance imaging; NF1, neurofibromatosis type 1. * At the time of diagnosis in an infant (1 month to 1 year). **Indicates to be performed in infants and children (1 year to adolescent). ***Advise for more frequent visits as indicated in children (5 years to adolescent). ^#^At least once in this time period in children (5 years to adolescent).

**Figure 3:**
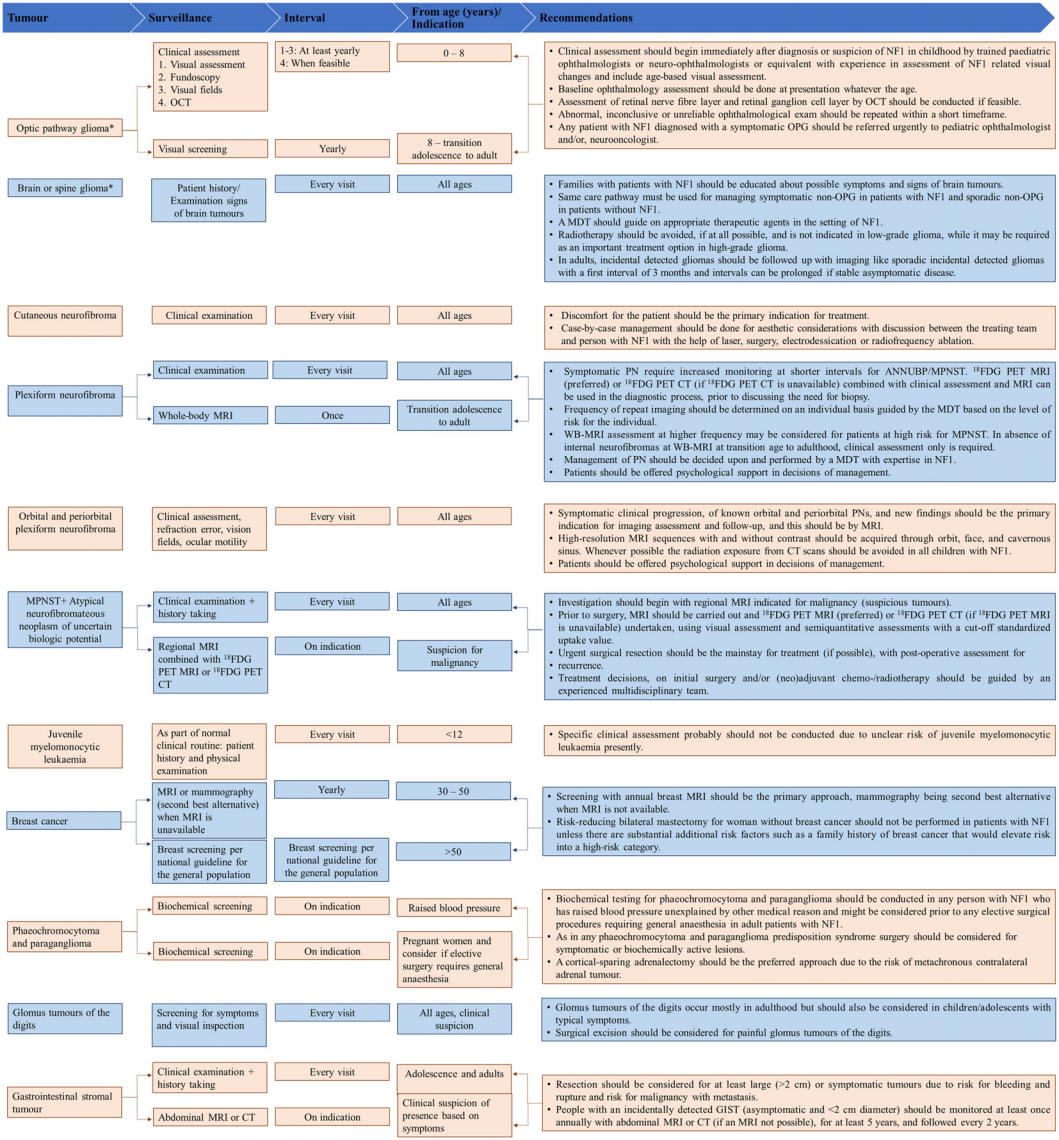
Overview of surveillance for tumor screening and recommendations for their management. Adapted from Carton et al., 2023 (75). CT, computed tomography; ^18^FDG PET MRI, ^18^F-fluorodeoxyglucose positron emission tomography magnetic resonance imaging; ^18^FDG PET-CT, ^18^F-fluorodeoxyglucose positron emission tomography-computed tomography; MEK, mitogen-activated protein kinase; MPNST, Malignant peripheral nerve sheath tumor; MRI, magnetic resonance imaging; mTOR, mammalian target of rapamycin; NF1, neurofibromatosis type 1; OCT, optic coherence tomography; PN, plexiform neurofibromas. *For high-grade gliomas, a detailed family history and genetic testing (whole exome sequencing) need to be taken if not already done.

**Figure 4:**
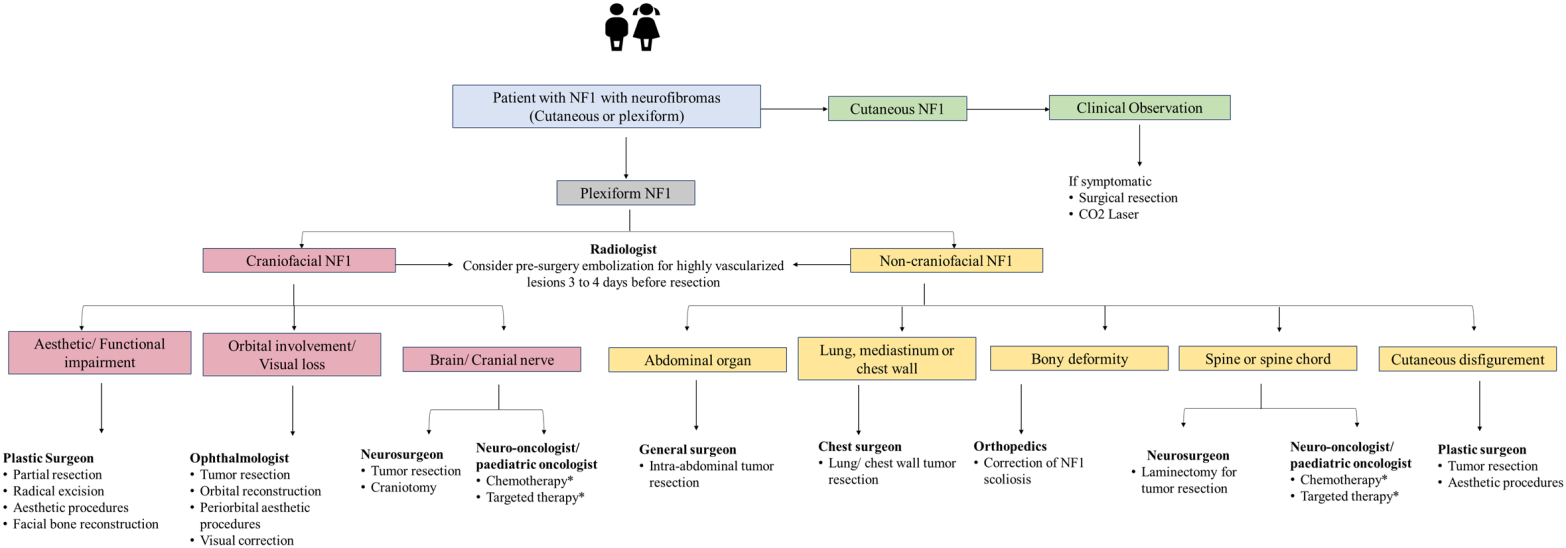
Management algorithm for NF1 associated with neurofibromas. NF1, neurofibromatosis type 1. *Treatment modality for unresectable plexiform neurofibroma and refractory tumor. Chemotherapy includes agents such as carboplatin-based therapy. Targeted therapy includes mitogen-activated protein kinase and mammalian target of rapamycin and MEK inhibitors.

**Figures 2**–**4** present an overview of the frequency of clinical and developmental assessment, surveillance for tumor screening, and management algorithms for NF1 (35, 75). Most studies recommend annual follow-up or as an as-needed approach in case of change or development of new indications (**Figures 2**, **3**) (1, 35, 75).

### 6.1 Neuroimaging

Imaging techniques, such as ^18^F-fluorodeoxyglucose (FDG) positron emission tomography (PET) MRI/CT, MRI, and CT along with clinical examination can be used for tumor screening in patients with NF1 (75). Currently, there are no consensus recommendations on appropriate intervals for plexiform neurofibroma monitoring using regional or WB-MRI. Factors such as age, tumor location, presence of plexiform neurofibroma-associated morbidity, imaging appearance, and prior imaging findings of any growth of the plexiform neurofibroma need to be considered to determine the scanning interval for WB-MRI. Few HCPs suggest performing WB-MRI at baseline (47), typically for patients who are transitioning from pediatric to adult care in late adolescence or early adulthood. Imaging intervals may be prolonged for those patients who have clinically or radiographically stable plexiform neurofibromas over time or reduced in case of new symptoms or changes in imaging appearance. Currently, 3D volumetric MRI tumor analysis is advised for clinical trials of plexiform neurofibromas due to its ability to reproducibly detect minor size changes (76, 77); but, accessibility is an issue with these 3D imaging tools (47). These imaging modalities can also be used to monitor patients, especially for assessing treatment responses. PET imaging is also a useful diagnostic adjunct for detecting malignant degeneration (78) and FDG-PET/CT is reported to have adequate diagnostic accuracy for detecting MPNST (79, 80).

The use of MRI screening is controversial for optical pathway glioma. Optic Pathway Task Force does not recommend the use of screening MRI (81), but studies have suggested that patients’ outcomes may be improved by early detection in young children (47, 73). Thus, it must be requested only in suspected cases of optic pathway gliomas (82). Studies have also shown that female NF1 patients with optic pathway gliomas had a higher likelihood of progressing and requiring treatment compared to male patients with the same surveillance (83). If a patient shows any indications or symptoms that suggest the presence of a new intracranial mass like increased intracranial pressure, a new neurologic deficit that may have its origin in the central nervous system, or a new onset of seizures, a brain MRI may be recommended (35).

### 6.2 Ophthalmic examinations

It includes visual acuity measurement, confrontation visual field testing, color vision assessment, evaluation of pupils, ocular motility, eyelids, fundi, and irises with a formal computerized or kinetic assessment of visual fields (as an adjunctive test in cases when the patient is reliable), optical coherence tomography to quantify the thickness of retinal nerve fiber layer (1). Visual evoked potential tests and/or imaging are suggested in case of equivocal results (1). Listernick et al. recommended that children with NF1 younger than 7 years should undergo yearly ophthalmic evaluations (84). Bergqvist et al. also suggested that all NF1-diagnosed children should be subjected to annual pediatric ophthalmological follow-up, at least till the age of 13 years (1). The panel from GCC recommended that a brain MRI must be requested only in suspected cases of optic pathway glioma. The panel also indicated for annual follow-up by a pediatric ophthalmologist or neuro-ophthalmologist.

### 6.3 Frequency and duration of follow-up

The panel from GCC indicated that height, weight, blood pressure, head circumference, physical examination of skin and spine, and visual function examinations are the most frequent assessment parameters used in the follow-up of patients with NF1. A follow-up period of 6 to 12 months is commonly practiced in the GCC region, however, newly diagnosed NF1 cases are advised to be followed every 6 months in order to assess the course or the evolution of the disease. Patients with a stable or inactive disease for many years were recommended to be followed annually. Macrocephaly and relative macrocephaly (disproportionately larger head size than height) typically do not require special follow-up (35). Thus, an annual spine assessment during childhood and early adolescence is needed in all patients and those with clinical evidence of scoliosis, and these patients must be referred to orthopedics (1). Bergqvist et al. suggested a follow-up of patients with MPNST that includes clinical examination and imaging, their frequency is based on tumor site and histological grade of tumor, with follow-up every three months for 3 years, every six months for the next 2 years followed by once annually (1).

### 6.4 Treatment of NF1

Treatment selection must be evaluated based on the suggestions from the MDT including surgeons and NF experts in medicine/pediatrics (47).

#### 6.4.1 Surgery

Removal of cutaneous neurofibromas may be recommended (in cases where they rub on clothing or cause discomfort) by a plastic surgeon or dermatologist. Based on the severity of pain, progression of neurological symptoms, and risk of permanent deficit, surgical intervention may be used to treat subcutaneous neurofibromas (85). Surgery is the first-line treatment for plexiform neurofibromas (86). Most experts on the panel also suggested that patients with plexiform neurofibroma are most commonly treated with surgery; however, the choice of surgical excision for plexiform neurofibroma varies from case to case. Experts also highlighted that the role of surgery as the preferred option for plexiform neurofibroma is still unclear due to the significant morbidity associated with surgery. It can frequently be challenging owing to the tumor’s pressure on nearby nerves and structures and its distinctive extensive vascularity, which may result in life-threatening hemorrhage (86).

#### 6.4.2 Radiation therapy

The scope of radiation therapy is limited for plexiform neurofibromas, especially stereotactic radiosurgery due to its indistinct borders, possibly resulting in difficulties in defining radiotherapy target volumes for highly conformal techniques (47).

For managing optic pathway gliomas, surgery may be less preferred due to tumor location; radiation may trigger secondary malignancies and vasculopathy (40). Radiotherapy and surgery are usually not recommended except in rare situations due to the increased risk of developing secondary malignancies, either gliomas, MPNST, or endocrine, neurovascular, and neuropsychological complications (87–89). Use of combination therapy with surgery and perioperative radiotherapy as the standard of care for high-grade, localized MPNSTs can significantly improve local control and survival outcomes compared to surgery alone (90). The decision to include radiotherapy as palliative therapy should be carefully considered based on the tumor characteristics.

#### 6.4.3 Chemotherapy

It remains the mainstay of treatment in patients with visual deterioration and/or radiologic progression. Chemotherapeutic agents such as carboplatin with and without vincristine, vinblastine, irinotecan, and avastin have been successfully used to treat optic pathway gliomas (91–94). The role of chemotherapy in MPNST is limited, especially in patients with NF1; the objective response rate ranges from 17.9% (NF1-related MPNST) to 44.4% (sporadic MPNST) (95). Doxorubicin-based regimens were the most commonly reported first-line chemotherapy regimens for advanced or metastatic MPNST (96). Ifosfamide is another chemotherapeutic agent which is commonly used and combination with doxorubicin has shown the best response compared to doxorubicin alone (97). Vincristine, cyclophosphamide, and dacarbazine have also been used in various combination chemotherapy regimens for MPNST, particularly in pediatric patients (96).

#### 6.4.4 Targeted therapies

The recent success of mitogen-activated protein kinase (MEK) inhibitors, which focus on a RAS downstream effector, is an addition to the landscape of plexiform neurofibromas management. Selumetinib, an oral, selective, MEK inhibitor, is used to treat patients with inoperable, symptomatic plexiform neurofibromas (98–103). Selumetibnib is approved in all GCC countries for the treatment of NF1-associated plexiform neurofibromas. A phase 1/2 trial evaluating the long-term safety and efficacy of selumetinib reported that more than two-thirds of the children with NF1-related plexiform neurofibromas had sustainable tumor shrinkage with up to 5 years of additional selumetinib treatment (102). A systematic review and meta-analysis showed an objective response rate of 73.8% (95% CI 57.3 to 85.5) and a disease control rate of 92.5% (95% CI 66.5 to 98.7) with the two most common adverse events of diarrhea and increased creatine kinase level (99). Other MEK inhibitors, mirdametinib, binimetinib, and trametinib are being investigated for the treatment of NF1-associated plexiform neurofibromas (104). Other MEK inhibitors, mirdametinib, binimetinib, and trametinib are being investigated for the treatment of NF1-associated plexiform neurofibromas (104, 105). Weiss et al. reported that 42% of patients treated with mirdametinib achieved a partial response and 53% had stable disease with a significant and durable decrease in pain ratings (106). Cabozantinib is a tyrosine kinase inhibitor, being assessed treatment of plexiform neurofibromas. A phase 2 clinical trial assessing the safety, efficacy, and biological activity of cabozantinib in adolescent patients with NF1 and progressive or symptomatic, inoperable plexiform neurofibromas reported that 42% of patients achieved a partial response with a significant reduction in tumor pain intensity (107). Acar et al. suggested that combining investigational agents such as MEK inhibitors and multi-tyrosine kinase inhibitors with surgical therapy may improve clinical response or increase time to tumor progression for plexiform neurofibromas (101). Additionally, the mammalian target of rapamycin (mTOR) and MEK inhibitors also may be considered for treating optic pathway gliomas but warrant more studies on efficacy and safety endpoints (108). Preliminary data from a phase I trial of the MEK inhibitor selumetinib trial showed that 40% with NF-associated optic pathway gliomas achieved partial response in selumetinib-treated patients and 37% had stable disease (109). Studies have also shown that bevacizumab-based chemotherapies were a well-tolerated and effective treatment for quick tumor control in order to preserve vision and improve morbidity (110, 111). Thus, there is a need for a personalized treatment plan for NF1 patients with optic pathway gliomas.

Most of the experts from the panel indicated prescribing MEK inhibitors in >50% of unresectable plexiform neurofibromas/low or high-grade glioma patients with NF1. Experts also suggested that although targeted therapy improves prognosis, only 50% of patients are responsive to this modality. The targeted therapy use is limited due to a lack of regional guidelines, unaffordability, and limited availability of these therapies for the management of plexiform neurofibromas in the NF1 population. Thus, individualized tailored treatment is the optimum choice to treat NF1 patients. Follow-up and management of other NF1 manifestations are presented in **Figures 2**–**4**.

## 7 Recommendations for referral pathways

**Figure 5:**
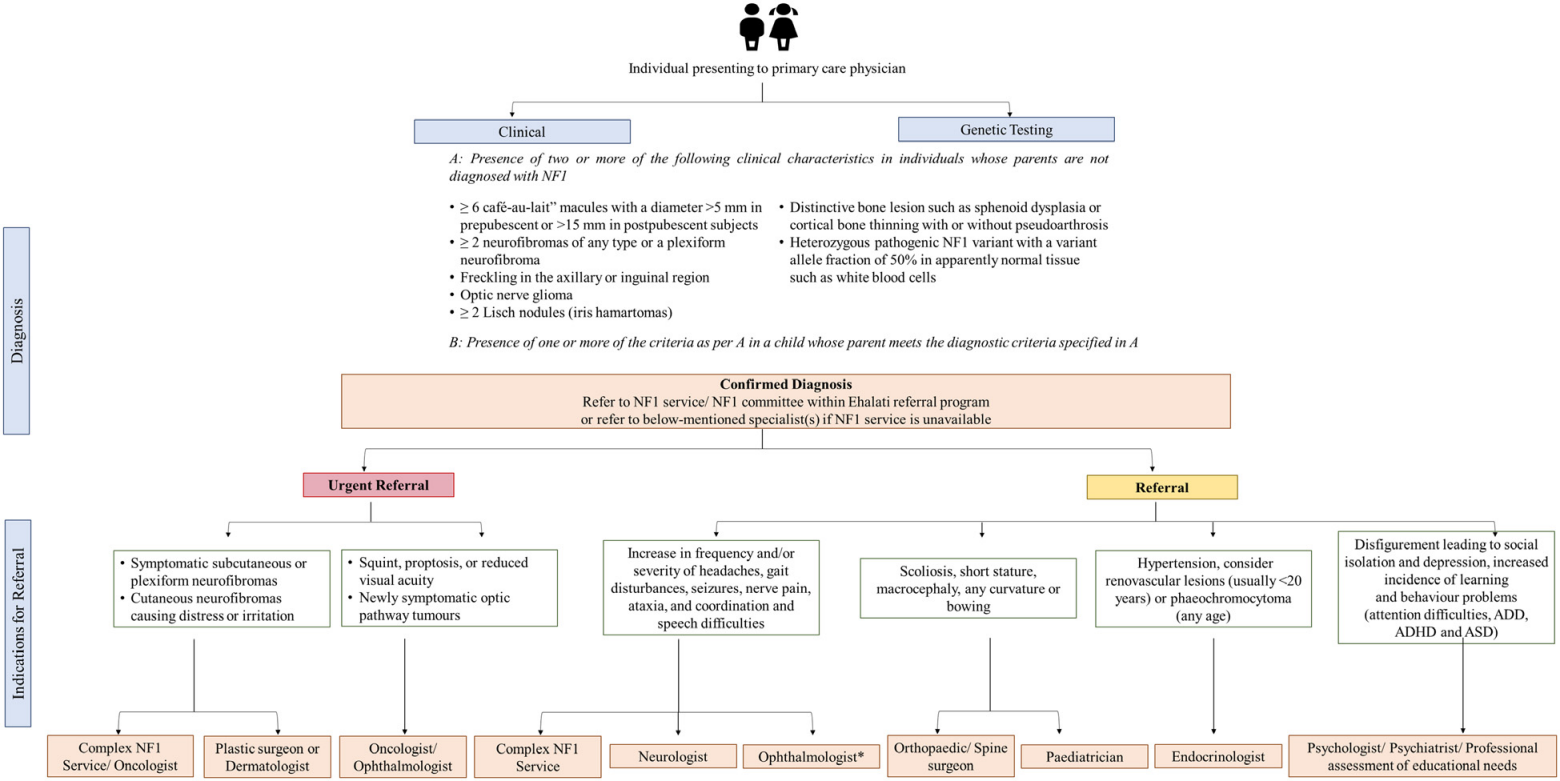
Proposed referral algorithm for patients with NF1. *All asymptomatic patients should be referred to an ophthalmologist. ADD, attention-deficit disorder; ADHD, attention-deficit/hyperactivity disorder; ASD, autism spectrum disorder; NF1, neurofibromatosis type 1; NIH, National Institutes of Health.

Depending on the patient’s age, natural history, clinical presentation, variability in NF1 manifestations, prognosis, complications, and warning signs, prompt and rapid medical attention for specialist care is needed for patients with NF1. Experts indicated urgent referral of patients with MPSNT and brain tumors, followed by optic pathway glioma and plexiform neurofibromas owing to their poor prognosis. In GCC, half of the experts indicated referring more than 50% of patients diagnosed with NF1 for genetic testing. Diagnosis of NF1 and other genetic diseases is a critical event in the child’s and parents’ lives. Regardless of whether genetic testing is done or not, patients and their families must be referred to a genetic counselor for a detailed discussion of clinical outcomes and future reproductive choices after procuring a detailed family history of a pediatric patient with NF1. Prenatal and pre-implantation genetic testing can be provided once the parent carrying the causative NF1 mutation has been found. However, as NF1 expressivity varies, it is frequently impossible to predict the severity of the disease. **Figure 5** shows the proposed referral algorithm for patients with NF1. Experts highlighted that indications for urgent and prompt referral to the concerned specialties are symptomatic subcutaneous or plexiform neurofibromas, cutaneous neurofibromas causing distress or irritation, squint, proptosis, reduced visual acuity or newly symptomatic optic pathway tumors. In such high-risk populations, a regular clinical evaluation must be performed at each visit that can be followed with a referral to apt specialists and oncologists as needed. Experts also pointed out that patients with visual symptoms must be urgently referred, while all asymptomatic patients must be referred to ophthalmology as routine referrals.

## 8 Recommendations for the multidisciplinary team and NF1 center of excellence

NF1 patients have an increased likelihood of developing both benign and malignant tumors. Due to the variable features and inherent clinical heterogeneity, HCPs need to be aware of the diverse clinical features of NF1 to provide prompt diagnosis and optimum care for patients with NF1. Additionally, clinical manifestations of NF1 starting in the early pediatric age require frequent long-term follow-ups by several specialists, underscoring the need for MDT care and setting up a multidisciplinary center to enhance the quality of care (13). A MDT includes surgeons (general, neurosurgeons, orthopedic surgeons, and plastic surgeons) and NF experts in medicine/pediatrics (oncologist, neuro-oncologist, and neurologist). Experts from the GCC region acknowledged that MDT was available in their centers, however, there was a noticeable difference in the percentage of patients with NF1 receiving treatment using the MDT approach.

**Figure 6:**
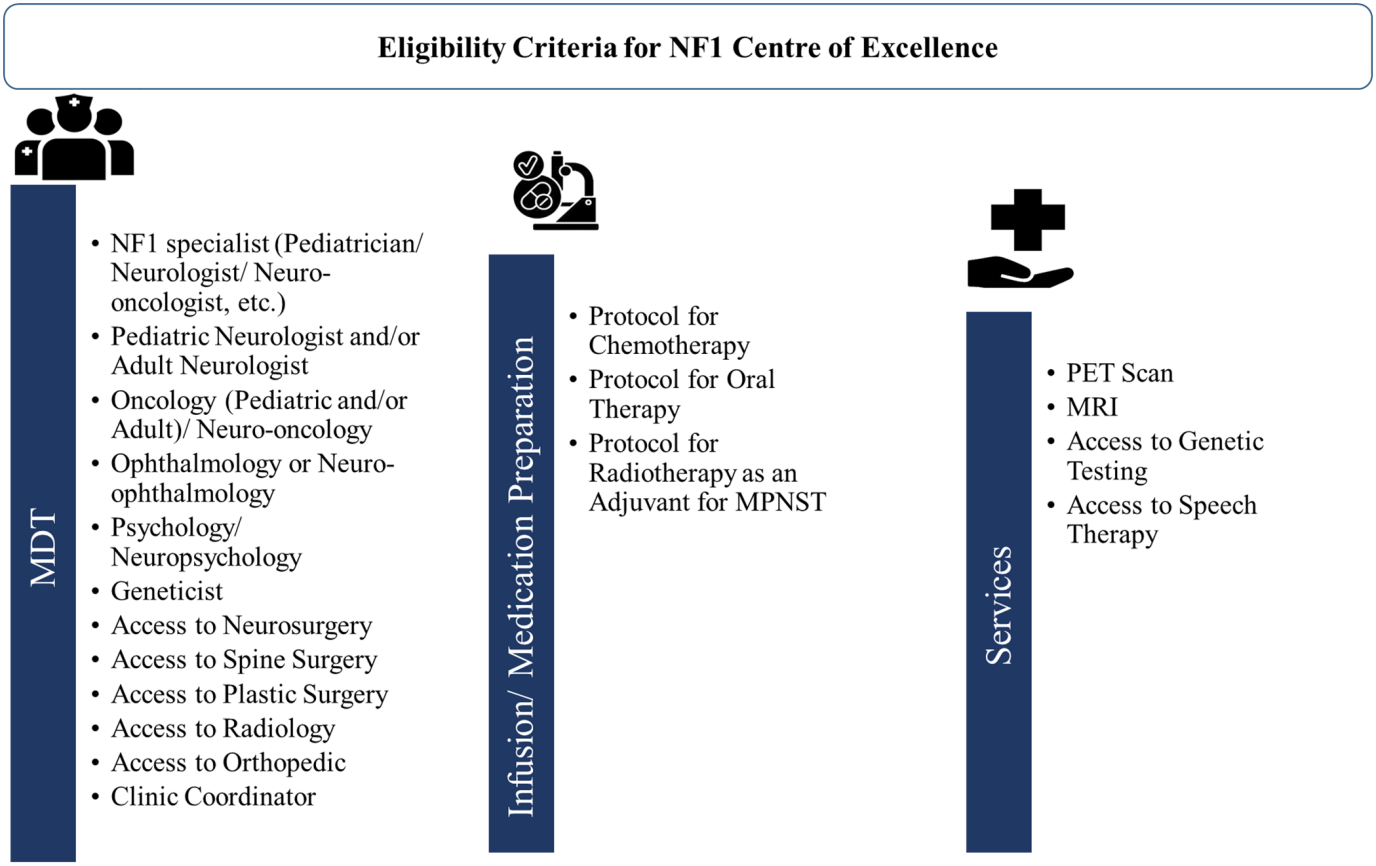
Overview of eligibility criteria for setting NF1 center of excellence. MDT, multidisciplinary team; MPNST, Malignant peripheral nerve sheath tumor; MRI, magnetic resonance imaging; NF1, neurofibromatosis type 1; PET, positron emission tomography.

A transition of NF1 patients from pediatric to adult care continues to be difficult due to the limited accessibility of specialty care for NF1 even in Western countries (112, 113). In the GCC region, the major barriers to developing MDT for NF1 care as discussed by experts were the lack of regional NF1 registries and regional guidelines, the lack of specialists in many regions, and the considerable time needed for MDT setup. To overcome these challenges, all the experts recommended setting up centers of excellence for NF1 at a regional level. They also advocated for developing easy referral pathways for primary care physicians, organizing virtual MDT meetings, and training as well as continued education programs for developing MDT in their region. **Figure 6** presents an overview of eligibility criteria for setting the NF1 center of excellence in the GCC region. Experts from GCC also underscore the need to evaluate the availability of MDT and services such as PET, MRI, and genetic testing in order to set up the center of excellence for NF1. A dedicated NF1 center of excellence will provide better accessibility to a broad range of specialties and recent treatment approaches through clinical trials. These clinics can also help in establishing registries on the prevalence and treatment of NF1. Improving the understanding of disease characteristics among patients, parents, and caregivers is a crucial component that can significantly impact the quality of life for individuals and families affected with NF1.

Several patient advocacy groups like Children’s Tumor Foundation, Littlest Tumor Foundation, Neurofibromatosis Network, and Children’s Hospital of Philadelphia are focused on creating awareness and disseminating knowledge to enhance the understanding of NF1 among patients and families affected by NF1 alongside driving the research for preventing therapies for NF1 (114–117). Online resources from these advocacy groups provide brochures on the cognitive and social development of children with NF1, genetic testing brochures, infographics with NF1 diagnostic criteria, optic pathway gliomas, vascular disease in NF1, videos discussing the neuropsychological evaluation of students, and a guide for patients with NF1 and caregivers transitioning to adult care (115, 118–120). These resources may help to provide a better understanding of NF1 and improve the quality of life among these patients. In the GCC, healthcare providers can also implement such initiatives in collaboration with the Saudi Pediatric Neurology Society or other societies by creating patient support groups and providing online educational resource materials for these patients to optimize the management of NF1.

## 9 Conclusion

NF1 is a complex genetic disease that often requires communication between experts from various specialists and lifetime monitoring of patients. Despite recent advances in the management of NF1, it is associated with significant morbidity owing to its complexity with a shorter lifespan compared to the general population. Pediatricians are crucial in the diagnosis and referral of patients because a majority of the principal NF1 manifestations appear in children or adolescents. Genetic testing is recommended for NF1 diagnosis, and a better insight into the genotype-phenotype correlation is required. In patients with NF1, surveillance with six months to annual follow-up is indicated for the development of new manifestations that require timely and prompt referral to the concerned specialist. For the effective management of patients with inoperable plexiform neurofibromatosis or low/high-grade optic pathway tumors, systemic therapy with MEK1/2 inhibitors is recommended. Thus, there is a need to optimize personalized NF1 care by adopting MDT-based treatment approaches. Establishing regional registries, and NF1 centers of excellence and developing regional management guidelines and referral pathways can pave the way for providing better care to patients with NF1 in the Arabian GCC region.
