# Corrigendum: Diagnosis and management of neurofibromatosis type 1 in Arabian Gulf Cooperation Council Region: challenges and recommendations

**DOI:** 10.3389/fonc.2024.1487777

**Published:** 2024-10-22

**Authors:** Fahad A. Bashiri, Khaled Hundallah, Musaad Abukhaled, Mossaed Mohammed Alyahya, Amna Al Futaisi, Daniah Alshowaeir, Asmaa Al Tawari, Shaker Abdullah, Ata Ur Rehman Maaz, Eman Taryam AlShamsi, Walaa Alshuaibi, Faisal Alotaibi, Hesham Aldhalaan

**Affiliations:** ^1^ Division of Pediatric Neurology, Department of Pediatrics, College of Medicine, King Saud University Medical City, King Saud University, Riyadh, Saudi Arabia; ^2^ Prince Sultan Military Medical City, Riyadh, Saudi Arabia; ^3^ Neuroscience Centre, King Faisal Specialist Hospital and Research Centre, College of Medicine, Al Faisal University, Riyadh, Saudi Arabia; ^4^ Consultant of Neuro-oncology and Neuromuscular-neurology, Department of Oncology, King Faisal Specialist Hospital and Research Centre, Department of Neuroscience, King Faisal Specialist Hospital and Research Centre, Al Faisal University, Riyadh, Saudi Arabia; ^5^ Sultan Qaboos University, College of Medicine and Health Sciences, Muscat, Oman; ^6^ Department of Ophthalmology, College of Medicine, King Saud University, Riyadh, Saudi Arabia; ^7^ Pediatric Neurology Unit, Pediatric Department, AlSabah Hospital, Ministry of Health, Kuwait City, Kuwait; ^8^ Department of Oncology, King Faisal Specialist Hospital & Research Center – Jeddah (KFSHRC-Jed), Jeddah, Saudi Arabia; ^9^ HemOnc Division, Department of Child Health, Sidra Medicine, Doha, Qatar; ^10^ Pediatric Hematology-Oncology Department, Al Jalila children’s specialty Hospital, Dubai, United Arab Emirates; ^11^ Division of Medical Genetics, Department of Pediatrics, King Khalid University Hospital, College of Medicine, King Saud University, Riyadh, Saudi Arabia; ^12^ Neuroscience Centre, King Faisal Specialist Hospital and Research Centre, Riyadh, Saudi Arabia; ^13^ Department of Neurosciences, King Faisal Specialist Hospital & Research Centre, Riyadh, Saudi Arabia

**Keywords:** children, Gulf Cooperation Council, management, neurofibromatosis type 1, referral

In the published article, there was an error in the FDA approval status of mirdametinib.

A correction has been made to section **6.4 Treatment of NF1**, subsection *6.4.4 Targeted therapies*, paragraph 1. This sentence previously stated:

“Mirdametinib is a recently approved MEK inhibitor in July 2023 for the management of NF1-associated plexiform neurofibromas (105).”

The corrected sentence appears below:

“Other MEK inhibitors, mirdametinib, binimetinib, and trametinib are being investigated for the treatment of NF1-associated plexiform neurofibromas (104, 105).”

The authors apologize for this error and state that this does not change the scientific conclusions of the article in any way. The original article has been updated.

