# Supplementary material for: Diagnosis and management of neurofibromatosis type 1 in Arabian Gulf Cooperation Council Region: challenges and recommendations
Source: Front Oncol. 2024 Aug 27;14:1323176. doi: 10.3389/fonc.2024.1323176 (PMC11385870; doi:10.3389/fonc.2024.1323176)
Supplement: Supplementary file 1 [file Table1.docx]

**Supplementary Material**

**Supplementary Table 1. NIH criteria for clinical diagnosis of NF1**

| **NIH Clinical Criteria*** | **Clinical Pearls** |
| --- | --- |
| ≥6 CALM, >5 mm in greatest diameter in prepubertal individuals and > 15 mm in greatest diameter in postpubertal individuals | - General population: 2 to 3 or fewer CALMs |
| Freckling in Axillary or inguinal regions | - General population: Freckling is unexpected in an area which is not exposed to the sun - Typically, it is not detected until the age of 5 or more |
| ≥2 neurofibromas of any type or one plexiform neurofibroma | - Usually, CALMs are present since birth in children with NF1 - Have smooth edges - Dermal and subcutaneous neurofibromas are not usually detected until later in childhood - Plexiform neurofibroma usually changes the texture and/ or colour of the overlying skin |
| Optic glioma | - Not detected without direct ophthalmoscopy - May be present in infants - Early detection is critical for preserving vision |
| ≥2 iris Lisch nodules (iris hamartomas) | - Age-related occurrence - rarely present in infants and toddlers - present in nearly half the school children and mostly in teenagers - Detected only with slit-lamp examination - Does not affect the vision of patients with NF1 |
| A distinctive osseous lesion such as sphenoid dysplasia or thinning of long bone cortex, with or without pseudarthrosis | - Tibial dysplasia ― the most common type of bone dysplasia - Tibial radiograph must be done in infants and toddlers with anterior-lateral tibial bowing and referred to orthopedics |
| First-degree relative (parent, sibling, or offspring) with NF1 by the above criteria | - NF1 shows 100% penetrance and does not skip a generation - Parents with NF1 must show some symptoms even if mildly affected |

CALM, café-au-lait macules; NIH, National Institutes of Health; NF1, neurofibromatosis type 1

*Two or more of the above criteria are required to establish the NF1 diagnosis (Source Leguis et al (26))

Note: A of conditions such as juvenile xanthogranulomas and nevus anemicus in the NIH clinical criteria might help improve early diagnosis of NF1 in younger children and infants with <2 diagnostic criteria (27,42).

***Pre-meeting Questionnaire***

1. **How many patients with NF1 do you see every month in your routine clinical practice?**

| ☐ <5  ☐ 11-20 | ☐ 6-10  ☐ >20 |
| --- | --- |

1. **Which criteria do you use for diagnosing NF1 in your clinical practice?**

| ☐ Clinical  ☐ Both | ☐ Genetic  ☐ Any other additional criteria, specify________________ |
| --- | --- |

1. **Which type of disease manifestations is commonly seen in patients with NF1 in your practice?**

| ☐ Café-au-lait macules (CALM)  ☐ Cutaneous neurofibromas  ☐ Short stature  ☐ Plexiform neurofibromas  ☐ Brain tumors  ☐ Optic pathway tumors  ☐ Malignant peripheral nerve sheath tumors | ☐ Axillary freckling  ☐ Scoliosis  ☐ Deafness  ☐ Lisch nodules  ☐ Macrocephaly  ☐ Learning disabilities  ☐ Hypertension  ☐ Other, specify________________ |
| --- | --- |

1. **In your opinion, which clinical manifestation results in a poor prognosis and requires prompt referral to a specialist?**

| ☐ CALM (Café-au-lait macules)  ☐ Cutaneous neurofibromas  ☐ Plexiform neurofibromas  ☐ Brain tumors  ☐ Optic pathway tumors  ☐ Scoliosis  ☐ Hypertension | ☐ Malignant peripheral nerve sheath tumors  ☐ Axillary freckling  ☐ Lisch nodules  ☐ Macrocephaly  ☐ Learning disabilities  ☐ Other, specify________________ |
| --- | --- |

1. **What percentage of patients with NF1 do you refer to the concerned specialities?**

| ☐ <5%  ☐ 16% - 50% | ☐ 5% - 15%  ☐ >50% |
| --- | --- |

1. **Do you strictly follow clinical guidelines (e.g. American College of Medical Genetics and Genomics, National Society of Genetic Counsellors) for diagnosis and management of NF1 in your practice?**

| ☐ Yes  ☐ Other (please specify) ______________ | ☐ No |
| --- | --- |

1. **In your practice, (a) how often do you follow-up with patients with NF1? (b) which NF1 assessment parameters do you use regularly? (Select all that apply)**

| **(a)**  ☐ 6 months  ☐ Once in a year  ☐ Once in 2 years  ☐ Once in 5 years | **(b)**  ☐ Height, weight, blood pressure, and head circumference  ☐ Physical examination of skin and spine  ☐ Ophthalmologic and cardiovascular examinations  ☐ Pubertal development  ☐ Speech and cognitive examinations  ☐ Other, specify________________ |
| --- | --- |

1. **Which type of burden is highest among patients with NF1 in your opinion? (Select all that apply)**

| ☐ Physical  ☐ Social | ☐ Economical  ☐ Psychological  ☐ Other, specify________________ |
| --- | --- |

1. **In which patients do you consider performing genetic testing? (Select all that apply)**

| ☐ All patients diagnosed with NF1 based on NIH criteria  ☐ Prenatal stage with identified parental mutation for NF1 | ☐ Patients with a family history of NF1 in a first-degree relative (parent, sibling, or child)  ☐ For differential diagnosis of NF1  ☐ Other, specify________________ |
| --- | --- |
| *Please provide your additional inputs* | |

1. **What percentage of patients do you refer for genetic testing and genetic counselling?**

| ☐ <5%  ☐ 16% - 50% | ☐ 5% - 15%  ☐ >50% |
| --- | --- |

1. **Which genetic mutations are frequently seen in your patients with NF1?**

| ☐ c1756-1759_delACTA mutation  ☐ NF1 microdeletion  ☐ TP53 mutations  ☐ NF1 Arg1276 variants  ☐ NF1 Arg1809  ☐ NF1 Met992del | ☐ KIAA1549:BRAF alteration  ☐ *NF1* 844–848 missense  ☐ NF1 Arg1038Gly  ☐ NF1 non-sense mutation in exon 13  ☐ Other, specify________________ |
| --- | --- |

1. **Which genetic mutations result in poor prognosis in patients with NF1? (Select all that apply)**

| ☐ c1756-1759_delACTA mutation  ☐ NF1 microdeletion  ☐ TP53 mutations  ☐ NF1 Arg1276 variants  ☐ NF1 Arg1809  ☐ NF1 Met992del | ☐ KIAA1549:BRAF alteration  ☐ *NF1* 844–848 missense  ☐ NF1 Arg1038Gly  ☐ NF1 non-sense mutation in exon 13  ☐ Other, specify________________  ☐ I am not sure |
| --- | --- |

1. **What are the most common treatment modalities used in your practice for managing plexiform neurofibroma? (Select all that apply)**

| ☐ Surgery  ☐ Chemotherapy | ☐ Radiotherapy  ☐ Other, specify________________ |
| --- | --- |

1. **According to your opinion, which targeted therapy has a better prognosis?**

| ☐ Tipifarnib  ☐ MEK 1/2 inhibitors (selumetinib/ trametinib) | ☐ TKIs (imatinib, sorafenib, cabozantinib)  ☐ mTOR inhibitors (sirolimus, everolimus)  ☐ Other, specify________________ |
| --- | --- |

1. **Are you comfortable with prescribing MEK inhibitor (Selumetinib) for patients with NF1 with inoperable plexiform neurofibromas / low or high-grade glioma?**

| ☐ Yes  ☐ Other, specify________________ | ☐ No |
| --- | --- |

1. **To what proportion of unresectable patients with NF1 with plexiform neurofibromas/ glioma would you prescribe MEK inhibitor?**

| ☐ <5%  ☐ 5% - 10%  ☐ 11% - 25% | ☐ 26% - 50%  ☐ >50%  ☐ None |
| --- | --- |

1. **Do you have a MDT clinic in your institution?**

| ☐ Yes | ☐ No |
| --- | --- |

1. **What percentage of patients with NF1 are discussed in MDT meetings in your clinical setting?**

| ☐ <5%  ☐ 11% - 25%  ☐ 26% - 50% | ☐ 5% - 10%  ☐ >50%  ☐ None |
| --- | --- |

1. **What challenges do you face in forming MDT for NF1 in your region? (Select all that apply)**

| ☐ Utilize considerable time  ☐ Lack of regional NF1 registries  ☐ Lack of regional guidelines | ☐ Lack of specialist care  ☐ Presence of clinical inertia  ☐ Other, specify________________ | |
| --- | --- | --- |
| *Please provide your additional inputs* | |  |

1. **What recommendations do you suggest for developing MDT in your region? (Select all that apply)**

| ☐ Provide easy referral pathways for primary care physicians  ☐ Develop training and continued education programs | ☐ Setting up centres of excellence for NF1 at a regional level  ☐ Organizing virtual MDT meeting  ☐ Other, specify________________ |
| --- | --- |

1. **Do you have any specific recommendations for the management of NF1 in your region?**

|  |
| --- |

We sincerely thank you for your time and efforts in completing the questionnaire. We may contact you if further information/clarification is required.
